# A Novel Lactate Metabolism-Related Gene Signature for Predicting Clinical Outcome and Tumor Microenvironment in Hepatocellular Carcinoma

**DOI:** 10.3389/fcell.2021.801959

**Published:** 2022-01-03

**Authors:** Yue Li, Huanye Mo, Shengli Wu, Xin Liu, Kangsheng Tu

**Affiliations:** ^1^ Department of Hepatobiliary Surgery, The First Affiliated Hospital of Xi’an Jiaotong University, Xi’an, China; ^2^ The Key Laboratory of Tumor Molecular Diagnosis and Individualized Medicine of Zhejiang Province, Zhejiang Provincial People’s Hospital, Affiliated People’s Hospital, Hangzhou Medical College, Hangzhou, China

**Keywords:** hepatocellular carcinoma, metabolic reprogramming, lactate, prognosis, tumor microenvironment

## Abstract

Hepatocellular carcinoma (HCC) is the main subtype of primary liver cancer with high malignancy and poor prognosis. Metabolic reprogramming is a hallmark of cancer and has great importance on the tumor microenvironment (TME). As an abundant metabolite, lactate plays a crucial role in cancer progression and the immunosuppressive TME. Nonetheless, the potential roles of lactate in HCC remain unclear. In this study, we downloaded transcriptomic data of HCC patients with corresponding clinical information from the TCGA and ICGC portals. The TCGA-HCC dataset used as the training cohort, while the ICGC-LIRI-JP dataset was served as an external validation cohort. Cox regression analysis and the LASSO regression model were combined to construct the lactate metabolism-related gene signature (LMRGS). Then, we assessed the clinical significance of LMRGS in HCC. Besides, enriched molecular functions, tumor mutation burden (TMB), infiltrating immune cells, and immune checkpoint were comprehensively analyzed in different LMRGS subgroups. In total, 66 differentially expressed lactate metabolism-related genes (LMRGs) were screened. The functions of LMRGs were mainly enriched in mitochondrial activity and metabolic processes. The LMRGS comprised of six key LMRGs (FKTN, PDSS1, PET117, PUS1, RARS1, and RNASEH1) had significant clinical value for independently predicting the prognosis of HCC patients. The overall survival and median survival of patients in the LMRGS-high group were significantly shorter than in the LMRGS-low group. In addition, there were differences in TMB between the two LMRGS subgroups. The probability of genetic mutations was higher in the LMRGS-high group. Most importantly, the LMRGS reflected the TME characteristics. In the LMRGS-high group, the immune microenvironment presented a suppressed state, accompanied by more inhibitory immune cell infiltration, including follicular helper T cells and regulatory T cells. Additionally, the expression of inhibitory checkpoint molecules was much higher in the LMRGS-high group. Our study suggested that the LMRGS was a robust biomarker to predict the clinical outcomes and evaluate the TME of patients with HCC.

## Introduction

Hepatocellular carcinoma (HCC) is the most common histological type of primary liver cancer, the third leading cause of cancer death worldwide (Sung et al., 2021). As a highly malignant tumor, the 5-year survival rate of HCC is less than 18% (Villanueva, 2019). Treatment options for HCC include hepatic resection, liver transplantation, image-guided ablation, transarterial therapies, chemotherapy, and molecularly targeted therapy (Llovet et al., 2021). Clinically, patients with HCC are often treated by a combination of several modalities. However, the therapeutic outcomes of advanced HCC remain unsatisfactory. Even after successful tumor eradication, the recurrence rate of HCC is remarkably high. Recently, immunotherapy has been shown to improve the clinical efficacy of advanced HCC. Unlike the mechanism of action of conventional therapy, immunotherapy is based on activating the patient’s own immune system to fight against tumors (Ringelhan et al., 2018). Cancer metabolism plays an essential role in affecting the anti-tumor immune response through modulating the interaction between tumor cells and the tumor microenvironment (TME) (Bader et al., 2020). Therefore, it is vital to identify a metabolism-related signature to assess the TME and improve the treatment efficacy of immunotherapy.

Metabolic alterations of tumor cells not only favor cell proliferation but also have profound influences on anti-tumor immunity through the release of metabolites, especially lactate (Xia et al., 2021). Unlike normal cells, tumor cells metabolize glucose to produce lactate even under adequate oxygen conditions. The accumulation of lactate provides an acidic microenvironment that benefits tumor growth and progression. Besides, lactate produced by aerobic glycolysis can be secreted into the extracellular environment as a signaling molecule to regulate intercellular interactions (Liao et al., 2021). In gastric cancer, lactate derived from tumor cells mediates the up-regulation of BDNF expression in cancer-associated fibroblasts by activating the NF-κB pathway, eventually resulting in acquired resistance (Jin et al., 2021). Alterations in lactate metabolism have been shown to be associated with cell invasion, migration, angiogenesis, drug resistance, and immune escape. High levels of lactate in the TME promote differentiation of tumor-associated macrophages to the M2 subtype, while activated macrophages facilitate tumor invasion through the CCL17/CCR4/mTORC1 signaling axis (Zhang et al., 2021). Lactate-induced PD-L1 up-regulation on neutrophils impairs T cell cytotoxicity in HCC (Deng et al., 2021). In addition, tumor cell-derived lactate induces the expression of GPR81 in dendritic cells via paracrine mode to inhibit the antigen presentation function of immune cells (Brown et al., 2020). Moreover, lactate has an important role in epigenetic regulation. Some studies have demonstrated that histone lysine lactylation takes part in modulating gene transcription (Izzo and Wellen, 2019; Yu et al., 2021). Given the vital role of lactate in oncogenesis and the immunosuppressive TME, targeting its metabolism promises to become an effective means for cancer treatment.

In this study, we screened the key lactate metabolism-related genes (LMRGs) and constructed a prognostic signature to predict the survival outcome. Next, we comprehensively analyze the tumor mutation burden (TMB) features in different subgroups. Then, the association between the TME and lactate metabolism-related gene signature (LMRGS) was explored using the R software package. We focused on the infiltrating immune cells in the TME and characterized the differential immune microenvironment in LMRGS subgroups. The results indicated that the LMRGS had a high value for evaluating the prognosis and reflecting the TME in HCC.

## Materials and Methods

### Data Acquisition

RNA transcriptome sequencing data, somatic mutation profile, and corresponding clinical information of HCC were obtained from the TCGA data portal (https://portal.gdc.cancer.gov/). In this study, the TCGA-HCC cohort was served as the training set. To verify the training set results, we downloaded an independent dataset of HCC from the ICGC website (https://dcc.icgc.org/releases/current/Projects/LIRI-JP). Therefore, the ICGC-LIRI-JP cohort was used as a validation set. The detailed clinical information of HCC patients from two cohorts was summarized in Table 1.

**TABLE 1:** Clinical and pathological characters of HCC patients in TCGA and ICGC cohort.

Characteristics		Number
TCGA cohort (*N* = 376)		
Age	≤60	180
	>60	196
Gender	Female	122
	Male	254
Pathological grade	G1	55
	G2	180
	G3	123
	G4	13
	NA	5
T	T1	185
	T2	94
	T3	81
	T4	13
	NA	3
N	N0	257
	N1	4
	NA	115
M	M0	272
	M1	4
	NA	100
Clinical Stage	I	175
	II	86
	III	86
	IV	5
	NA	24
Fibrosis	No	76
	Yes	141
	NA	159
Virus infection	No	210
	Yes	166
Vascular invasion	No	210
	Yes	110
	NA	56
ICGC cohort (*N* = 231)		
Age	≤60	49
	>60	182
Gender	Female	61
	Male	170
Clinical Stage	I	36
	II	105
	III	71
	IV	19

### Differentially Expressed LMRGs and Transcription Factors

The 289 LMRGs were retrieved from the Molecular Signatures database (Liberzon et al., 2015). Transcription factors associated with cancer were downloaded from the Cistrome (Zheng et al., 2019). To identify the LMRGs and transcription factors involved in the progression of HCC, we carried out differential expression analysis between 50 normal tissues and 374 tumor tissues in the TCGA-HCC cohort. Genes with |log2 fold change (FC) | > 1 and false discovery rate (FDR) < 0.05 were defined as differentially expressed. For further understanding the biological function and pathway of differentially expressed LMRGs and transcription factors, we used the “clusterprofiler” package in R (version 4.1.0) to carry out the GO and KEGG enrichment analyses.

### Construction and Assessment of LMRGS

The differentially expressed LMRGs were subjected to univariate Cox regression analysis to determine the LMRGs with prognostic value. To avoid overfitting, we further performed the LASSO Cox regression (iteration = 1000) using the “glmnet” package (Friedman et al., 2010; Liu et al., 2021a; Liu et al., 2021b). After screening by LASSO regression, the selected LMRGs were applied to establish the LMRGS through the multivariate Cox regression analysis. The LMRGS score was calculated as the following formula: LMRGS score = expression level of gene_1_ × coefficient of gene_1_ + expression level of gene_2_ × coefficient of gene_2_ + … + expression level of gene_n_ × coefficient of gene_n_. We classified HCC patients into two subgroups according to the median LMRGS score, including the LMRGS-high and the LMRGS-low groups. Principal component analysis (PCA) was used to evaluate the classification accuracy of the signature. For assessing the prognostic value of the LMRGS, we conducted the Kaplan–Meier (KM) survival analysis to compare the overall survival (OS) and median survival time between the two LMRGS groups. The time-dependent ROC curve was performed by the “timeROC” package in R. We also applied the Cox proportional hazards regression model to identify the LMRGS as an independent predictor for OS. To explore the influence of the LMRGS on HCC progression, we clarify the association between the LMRGS and clinicopathologic factors, such as TNM stage, pathological grade, fibrosis, vascular invasion, and virus infection.

### Establishing a Nomogram

To predict the one-, three-, 5-year survival rate of HCC patients, we constructed a nomogram based on the LMRGS and significant clinicopathologic parameters (Iasonos et al., 2008). The calibration curve was used to estimate the consistency between predicted survival and actual survival. The time-dependent ROC curve was applied to evaluate the specificity and sensitivity of the model.

### Calculation of TMB

For calculating the TMB of each HCC tumor sample, we selected the somatic mutation data processed by the VarScan platform in the TCGA-HCC cohort. Then, we compared the difference of TMB between the LMRGS-high and the LMRGS-low groups. Visualization of somatic mutations in the two LMRGS groups was performed by the R package “maftools”. Moreover, we explored the impact of the LMRGS score combined with the TMB on the survival of HCC.

### Comprehensive Analysis of TME in Different LMRGS Subgroups

The TME is mainly composed of stromal cells and immune cells (Gysler and Drapkin, 2021). Firstly, we used the ESTIMATE algorithm to calculate the stromal score of all samples (Yoshihara et al., 2013). ESTIMATE is a prevalent R package, which is widely utilized in the cancer-related studies (Liu et al., 2021c; 2021d; 2021e). Then, the single sample gene set enrichment analysis (ssGSEA) was performed to derive the immune enrichment score based on the 29 immune gene sets (Bindea et al., 2013). To identify the immune infiltration features of HCC samples, we imported their gene expression profiles to the CIBERSORTx website with 1000 permutations (https://cibersortx.stanford.edu/). According to the obtained results, we compared the relative fractions of 22 tumor-infiltrating immune cells in the two LMRGS subgroups. Moreover, correlation analysis was carried out to clarify the relationship between the immune cell and the LMRGS score. Immune checkpoints expression and immune function have crucial influences on the treatment responses of immunotherapy. For further investigating the effect of the LMRGS score on immunotherapy, comparisons between the two LMRGS subgroups were analyzed to evaluate the differences of immune checkpoints and immune function.

### Gene Set Enrichment Analysis

The HCC samples were stratified into high- and low-LMRGS score groups as described above. To determine the primary signaling pathways and hallmark gene sets involved in the signature, we uploaded sample grouping and gene expression files into the GSEA software (version 4.1.0) to conduct enrichment analysis.

### Statistical Analysis

All data analysis and visualization were completed by R software. If the data did not follow a normal distribution and the variance was uninformed, the differences between groups were compared by the Wilcoxon rank-sum test or Kruskal–Wallis test. The Cox regression model was used to perform univariate and multivariate analyses. The log-rank test was performed to evaluate the survival difference. Correlation analyses of LMRGS score and immune infiltration cells were conducted by Spearman’s rank correlation test. In this study, *p*-value < 0.05 was considered statistically significant as indicated.

## Results

### Identification of LMRGs

**FIGURE 1:**
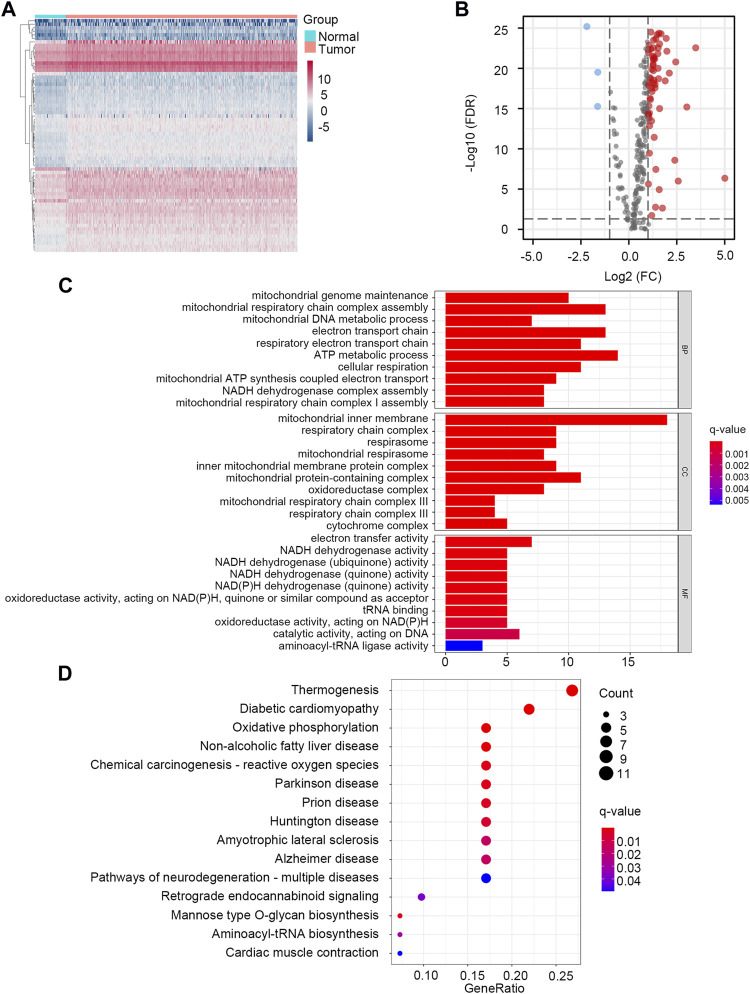
Identification and enrichment analysis of LMRGs in HCC. **(A)** The heatmap showed the expression level of LMRGs in each sample. **(B)** The volcano plot displayed down-regulated and up-regulated LMRGs. **(C)** GO enrichment analysis. **(D)** KEGG pathway enrichment analysis.

Through the differential gene screening analysis, we obtained 66 differentially expressed LMRGs, including three down-regulated and 63 up-regulated genes. The heat map displayed the expression of LMRGs in HCC samples and normal samples (Figure 1A). The differential expression of down-regulated and up-regulated LMRGs was represented in the volcano plot (Figure 1B). The 66 differentially expressed LMRGs were further analyzed by functional enrichment analysis. The primary biological processes (BP) of LMRGs were involved in mitochondrial genome maintenance, mitochondrial respiratory chain complex assembly, electron transport chain, and metabolic process. For cellular components (CC), the LMRGs primarily existed in the mitochondrial inner membrane, respiratory chain complex, and mitochondrial respirasome. The molecular functions (MF) of LMRGs were mainly enriched in electron transfer activity, NADH dehydrogenase activity, and oxidoreductase activity (Figure 1C). Signaling pathway analysis indicated that the differentially expressed LMRGs were related to thermogenesis, diabetic cardiomyopathy, oxidative phosphorylation, non-alcoholic fatty liver disease, and reactive oxygen species (Figure 1D). The above results showed that the LMRGs were mainly associated with metabolic processes and oxidation responses.

### Development of the LMRGS

**FIGURE 2:**
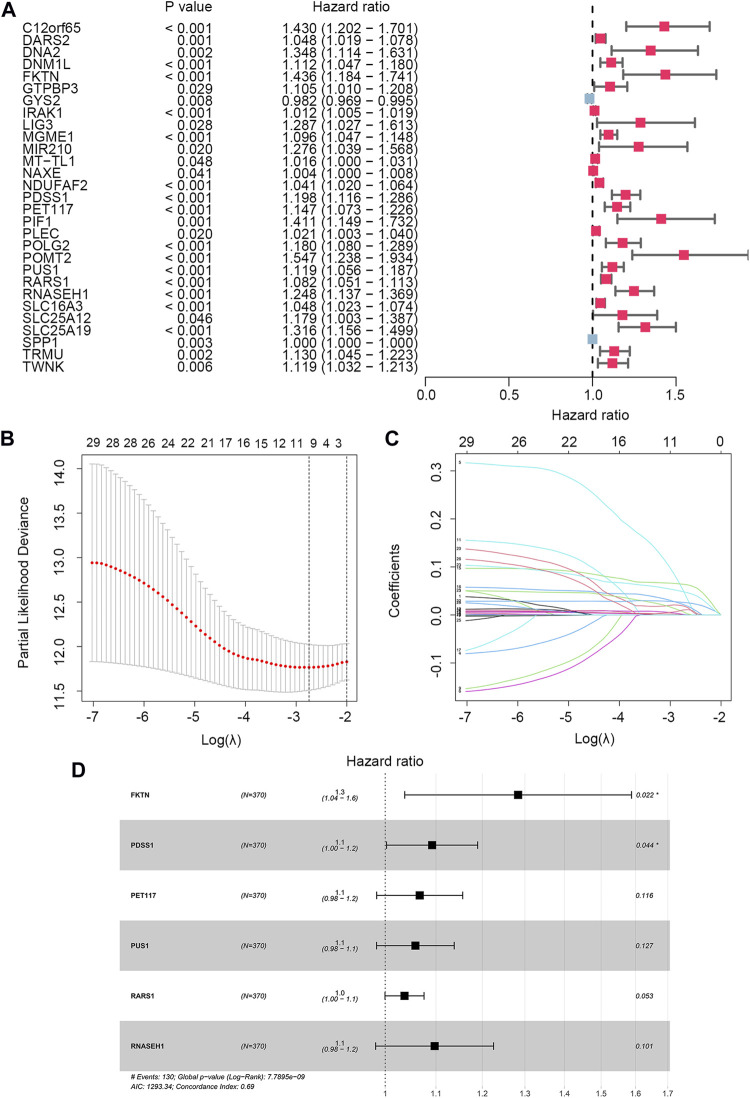
Cox regression analysis and LASSO analysis of LMRGs. **(A)** Univariate Cox regression analysis screened 29 prognostic LMRGs. **(B)** Tuning parameter (*λ*) selection in LASSO model using cross-validation. **(C)** The LASSO coefficient profile of 29 prognostic LMRGs. **(D)** Multivariate Cox regression analysis of LMRGs was shown by forest plot.

To identify the LMRGs correlated with OS, we performed the univariate Cox regression analysis. A total of 29 LMRGs were related to prognosis (Figure 2A). After selection by LASSO regression, only 10 LMRGs were subjected to multivariate Cox regression analysis to construct the LMRGS (Figures 2B,C). Based on the coefficient and the expression of six crucial genes involved in the LMRGS, we calculated the LMRGS score (Figure 2D). The LMRGS score of every HCC patient was obtained as follows: LMRGS score = FKTN expression × 0.2496 + PDSS1 expression × 0.0881 + PET117 expression × 0.0648 + PUS1 expression × 0.0567 + RARS1 expression × 0.0362 + RNASEH1 expression × 0.0928.

### Prognostic Significance of the LMRGS

**FIGURE 3:**
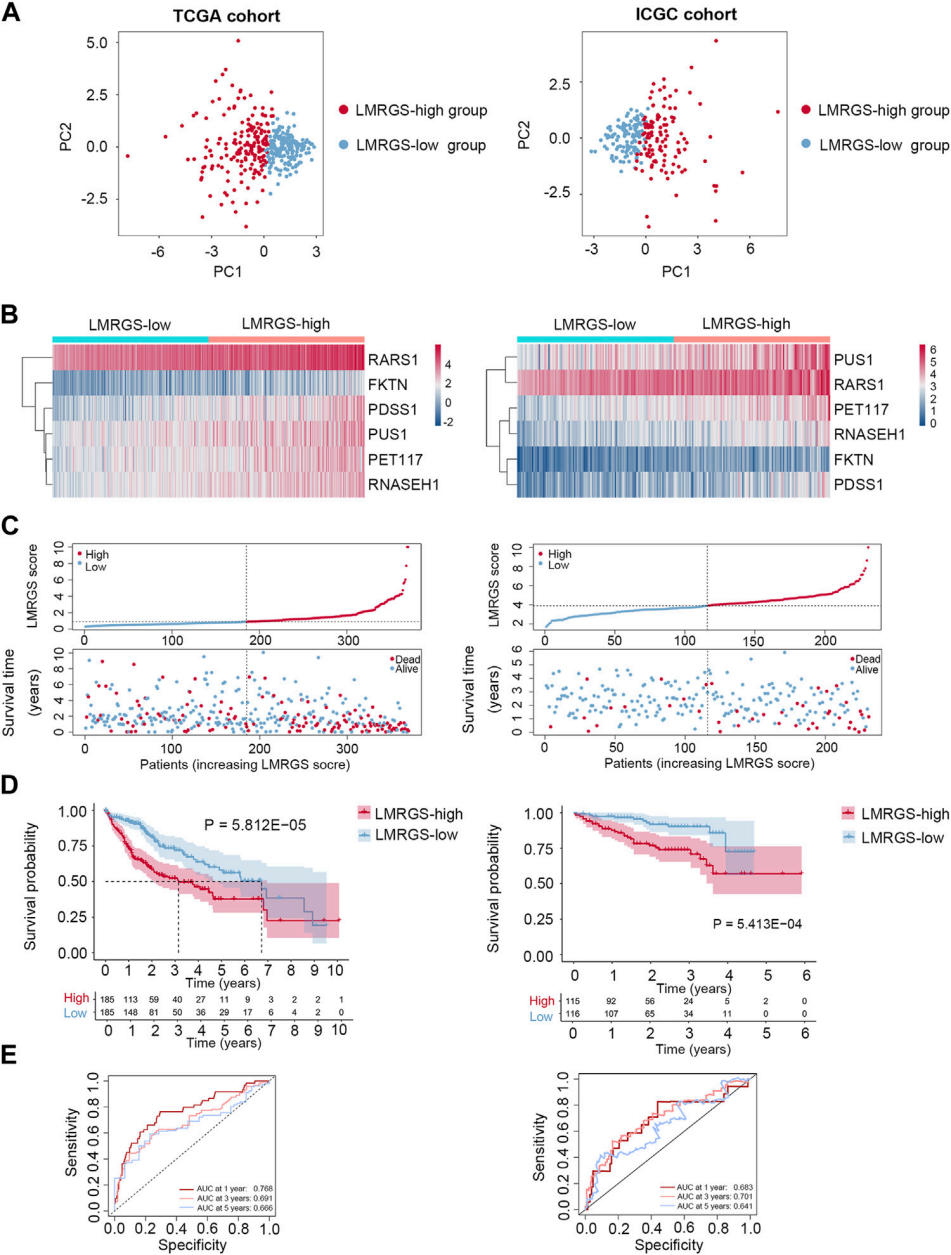
Prognostic value of LMRGS in HCC. **(A)** PCA was used to determine whether the samples could be grouped correctly based on the LMRGS score. **(B)** Heatmap for the expression of six crucial genes in LMRGS-low and LMRGS-high groups. **(C)** The distribution of LMRGS scores and survival status of HCC patients with increasing LMRGS score. **(D)** KM survival analysis between LMRGS-low and LMRGS-high groups. **(E)** ROC curves analysis of LMRGS on OS at 1 year, 3 years, and 5 years.

Taking the median LMRGS score as cut-off, we divided the HCC patients into two subgroups: LMRGS-high and LMRGS-low groups (Figure 3A). The heat map showed the differential expression of six crucial genes in the two LMRGS subgroups (Figure 3B). The LMRGS score and survival status of every HCC patient were displayed in Figure 3C. KM analysis indicated that patients with the high LMRGS score had shorter OS and median survival than patients with the low LMRGS score (Figure 3D). According to the different clinical characteristics, subgroup survival analysis also confirmed this result (Supplementary Figure S1). As shown in Figure 3E, the area under curve (AUC) value of 1 year, 3 years, and 5 years for ROC analysis was 0.768, 0.691, and 0.666, respectively, in the TCGA cohort. Moreover, the univariate and multivariate regression analyses demonstrated that the LMRGS score was an independent risk factor for OS (HR = 3.576, 95%CI = 2.105–6.074, *p* = 2.44E-06) (Table 2). The correlation of the LMRGS score and clinicopathological factors was clarified in the TCGA cohort. The results suggested that the LMRGS score was closely associated with pathological grade, clinical stage, vascular invasion, and virus infection (Supplementary Figure S2). The above results indicated that the LMRGS score played a vital role in HCC progression.

**TABLE 2:** Univariate and multivariate Cox regression analyses of the LMRGS score in the TCGA.

Variable	Univariate analysis			Multivariate analysis		
	HR	95% CI	*p*-value	HR	95% CI	*p*-value
LMRGS score	3.461	2.179–5.499	1.469E-7	3.576	2.105–6.074	2.44E-6
Age	1.015	0.993–1.037	0.177	1.015	0.993–1.039	0.189
Gender	0.574	0.335–0.982	0.043	0.851	0.446–1.624	0.625
Grade	1.235	0.847–1.799	0.273	1.247	0.808–1.925	0.318
Clinical Stage	1.692	1.273–2.249	0.000	1.374	0.300–6.283	0.682
T	1.616	1.229–2.125	0.001	0.950	0.235–3.847	0.943
N	2.983	0.410–21.704	0.280	0.663	0.017–25.662	0.826
M	4.895	1.515–15.819	0.008	3.349	0.711–15.767	0.126
Fibrosis	0.589	0.335–1.035	0.066	0.875	0.468–1.636	0.675
Virus infection	2.252	1.310–3.872	0.003	1.923	1.050–3.552	0.034
Vascular invasion	1.330	0.760–2.329	0.317	0.828	0.451–1.517	0.540

HR, hazard ratio; 95%CI, 95% confidence interval.

### A Nomogram for Predicting Survival

**FIGURE 4:**
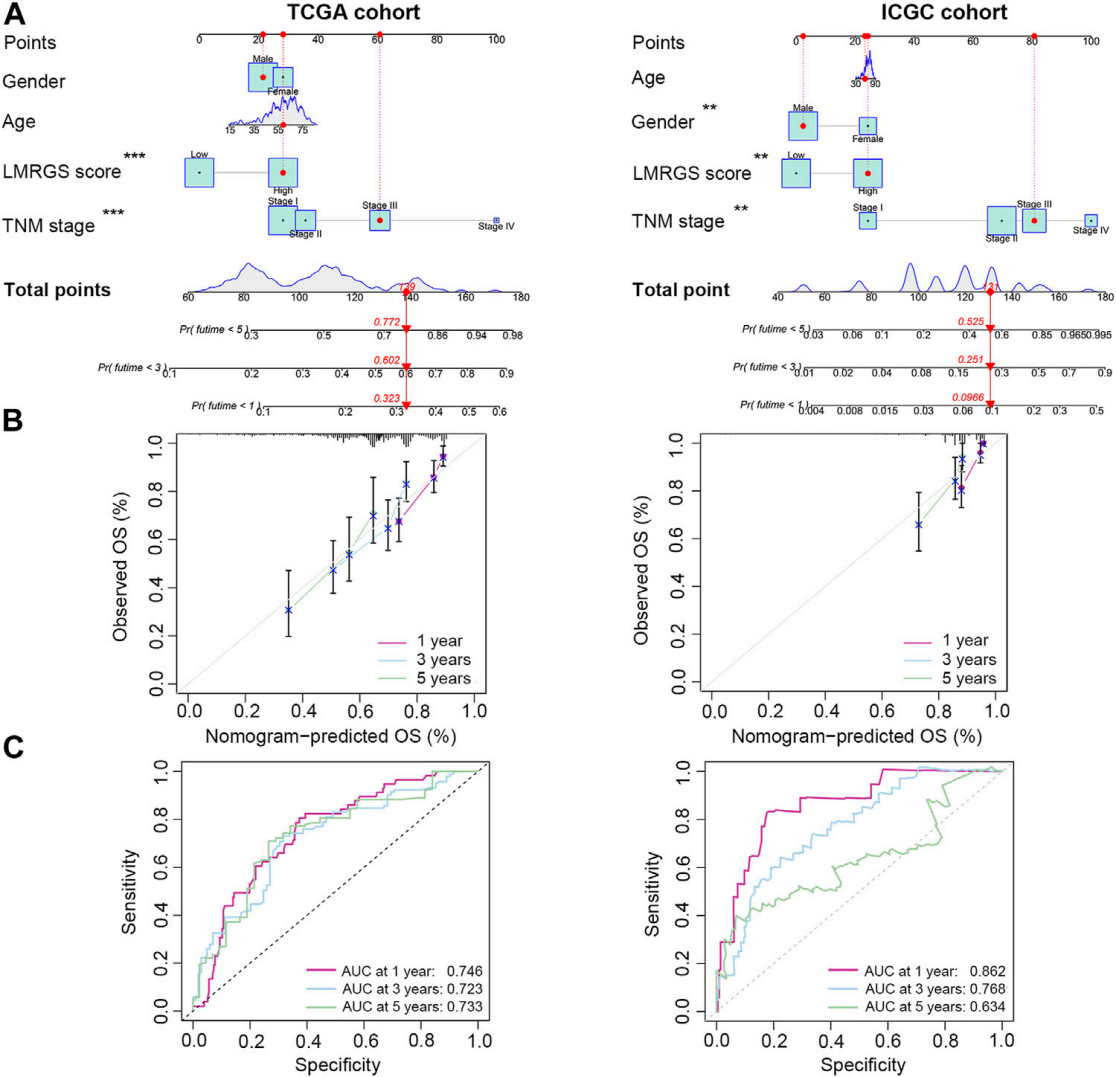
A nomogram was generated to estimate the survival rate of HCC patients. **(A)** Development of a nomogram by combining LMRGS score with age, gender, and TNM stage to predict the survival probability. **(B)** Calibration plots of the nomogram. **(C)** ROC curves of the nomogram. *p < 0.05, **p < 0.01, ***p < 0.001.

To accurately predict the probability of OS, we established a nomogram that integrated the LMRGS score and other clinicopathological features, including age, gender, and TNM stage (Figure 4A). We could estimate the survival rate of 1 year, 3 years, and 5 years based on the total points. The calibration curve demonstrated that the prediction value was highly consistent with the actual value (Figure 4B). The time-dependent ROC curve also indicated that this nomogram had high accuracy for predicting survival (Figure 4C).

### Regulation Network of Transcription Factors

**FIGURE 5:**
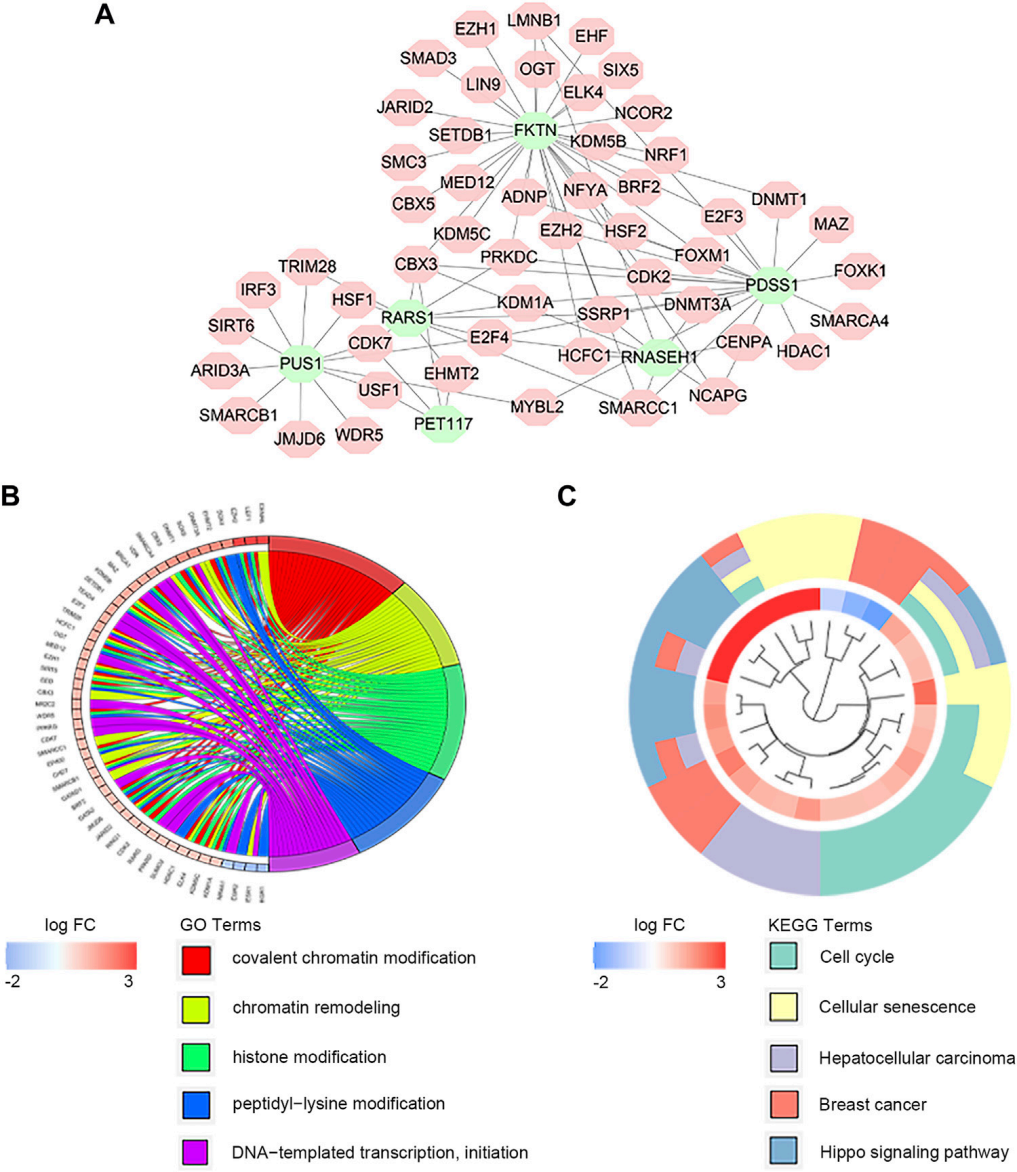
Co-expression of transcription factors and key LMRGs. **(A)** Regulatory network of key LMRGs and transcription factors. **(B)** GO enrichment results of transcription factors. **(C)** KEGG enrichment results of transcription factors.

There exist close interactions between the LMRGs and transcription factors. For exploring the relationship, we carried out the co-expression analysis. As displayed in Figure 5A, we identified 52 differential expressed transcription factors co-expressed with six significant LMRGs. The main functions of co-expressed transcription factors were chromatin remodeling and histone modification (Figure 5B). KEGG analysis revelated that these transcription factors mainly participated in the cell cycle, cellular senescence, and Hippo signaling pathway (Figure 5C).

### Association With TMB

**FIGURE 6:**
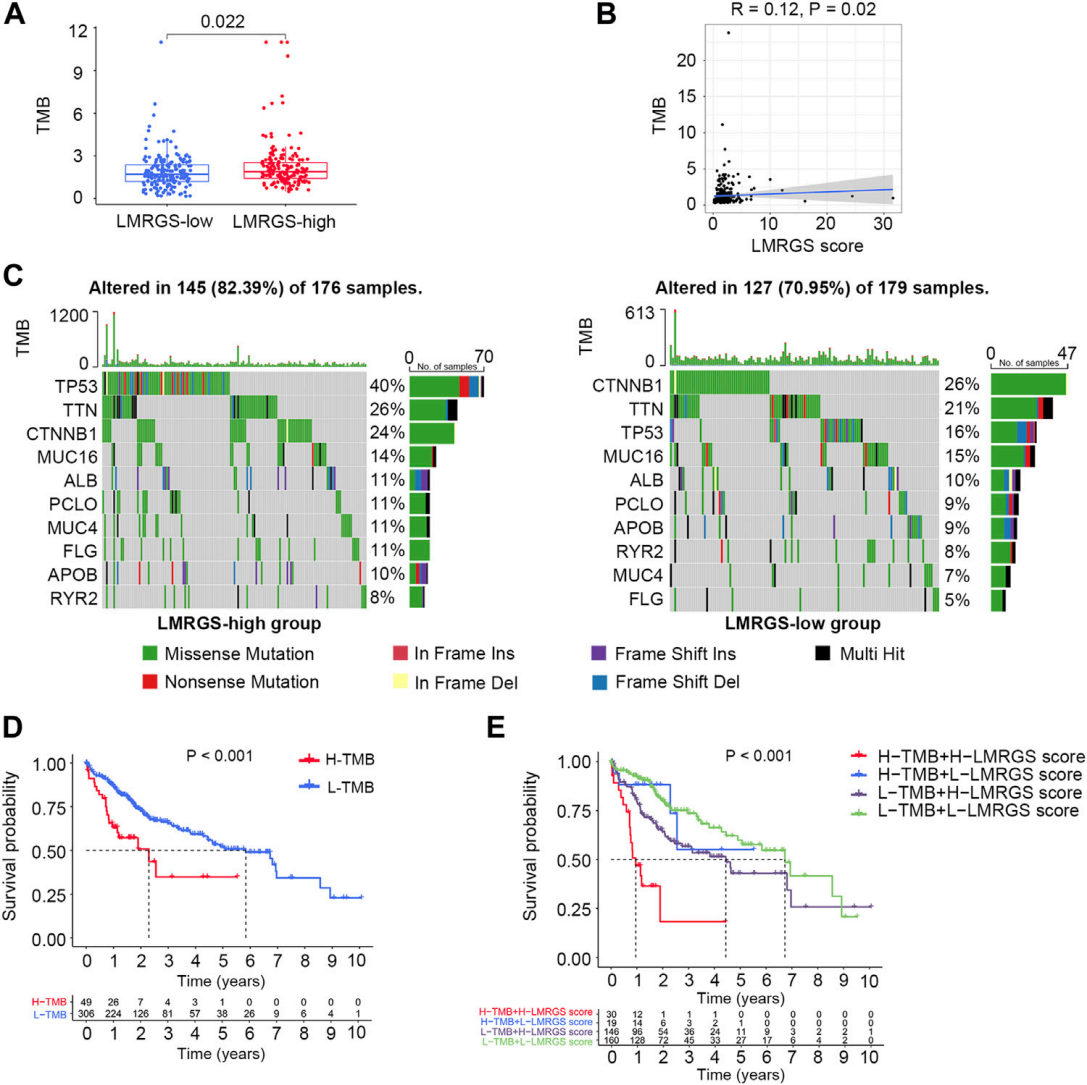
Tumor mutation characteristics in different LMRGS subgroups. **(A)** The differences of TMB in LMRGS-low and LMRGS-high groups. **(B)** The association of TMB with LMRGS score. **(C)** Top 10 mutated genes in different LMRGS subgroups. **(D)** KM survival analysis of TMB. **(E)** Effects of the LMRGS score combined with TMB on the overall survival.

In the TCGA training cohort, we calculated the TMB of each HCC patient. We found that the TMB was higher in the LMRGS-high group (Figures 6A,B). Then, mutant situations of different LMRGS subgroups were visualized by the waterfall plots (Figure 6C). For the entire dataset, the top 10 mutated genes in HCC were TP53, CTNNB1, TTN, MUC16, ALB, PCLO, APOB, RYR2, MUC4, and FLG. Missense mutations were the most common somatic mutational types. The mutation frequency of samples was higher in the LMRGS-high group. Moreover, patients with high LMRGS scores had a higher mutation probability of crucial genes, especially TP53. Subsequently, we performed KM analysis to evaluate the influence of the LMRGS score combined with the TMB on survival. The result showed that the survival time of the high-TMB group was shorter than the low-TMB group (Figure 6D). More importantly, patients with a low LMRGS score and low TMB had a significantly longer OS than patients with a high LMRGS score and high TMB (Figure 6E). In the ICGC validation cohort, we also analyzed the mutation profiles of all samples. There existed no TMB difference among the two LMRGS subgroups (Supplementary Figures S3A,B). However, the mutation frequencies of prevalently mutated genes in HCC were higher in the LMRGS-high group (Supplementary Figure S3C). Survival analysis results of the LMRGS score combined with the TMB were consistent with the training cohort (Supplementary Figures 3D,E).

### TME Characteristics in Different LMRGS Subgroups

**FIGURE 7:**
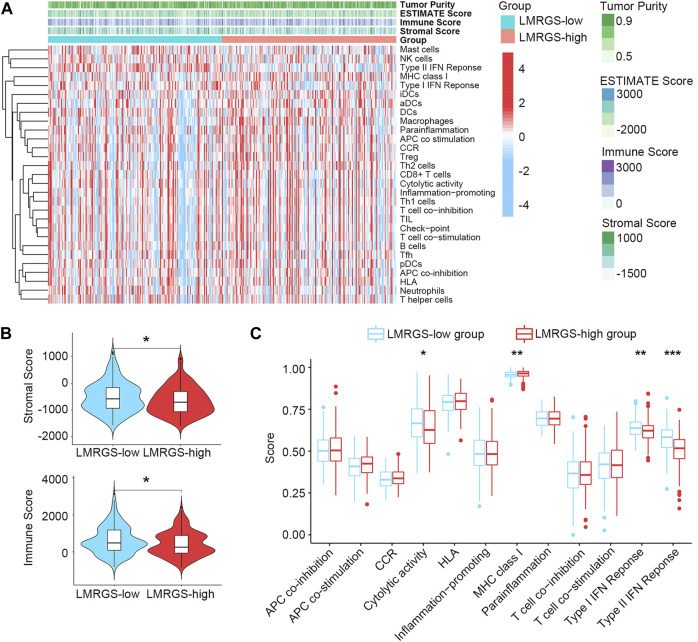
The landscape of TME in HCC. **(A)** Stromal score and immune activity of all HCC samples. **(B)** The violin plot showed the difference in stromal scores and immune scores between LMRGS-low and LMRGS-high groups. **(C)** Differences in immune function between the two subgroups. *p < 0.05, **p < 0.01, ***p < 0.001.

Stromal cells and immune cells in the TME have profound impacts on tumor progression, treatment efficacy, and clinical outcomes. The heatmap shown in Figure 7A and Supplementary Figure S4A displayed the stromal score and immune activity of all samples. We found that the abundance of stromal cells was relatively higher in the LMRGS-low group. In addition, the LMRGS-low group had higher immune scores than the LMRGS-high group (Figure 7B and Supplementary Figure S4B). As shown in Figure 7C and Supplementary Figure S4C, there were differences in immune function between the LMRGS-high and LMRGS-low groups. The activity of cytolysis and IFN response was higher in the LMRGS-low group. In the LMRGS-high group, there was a higher expression of MHC class Ⅰ.

**FIGURE 8:**
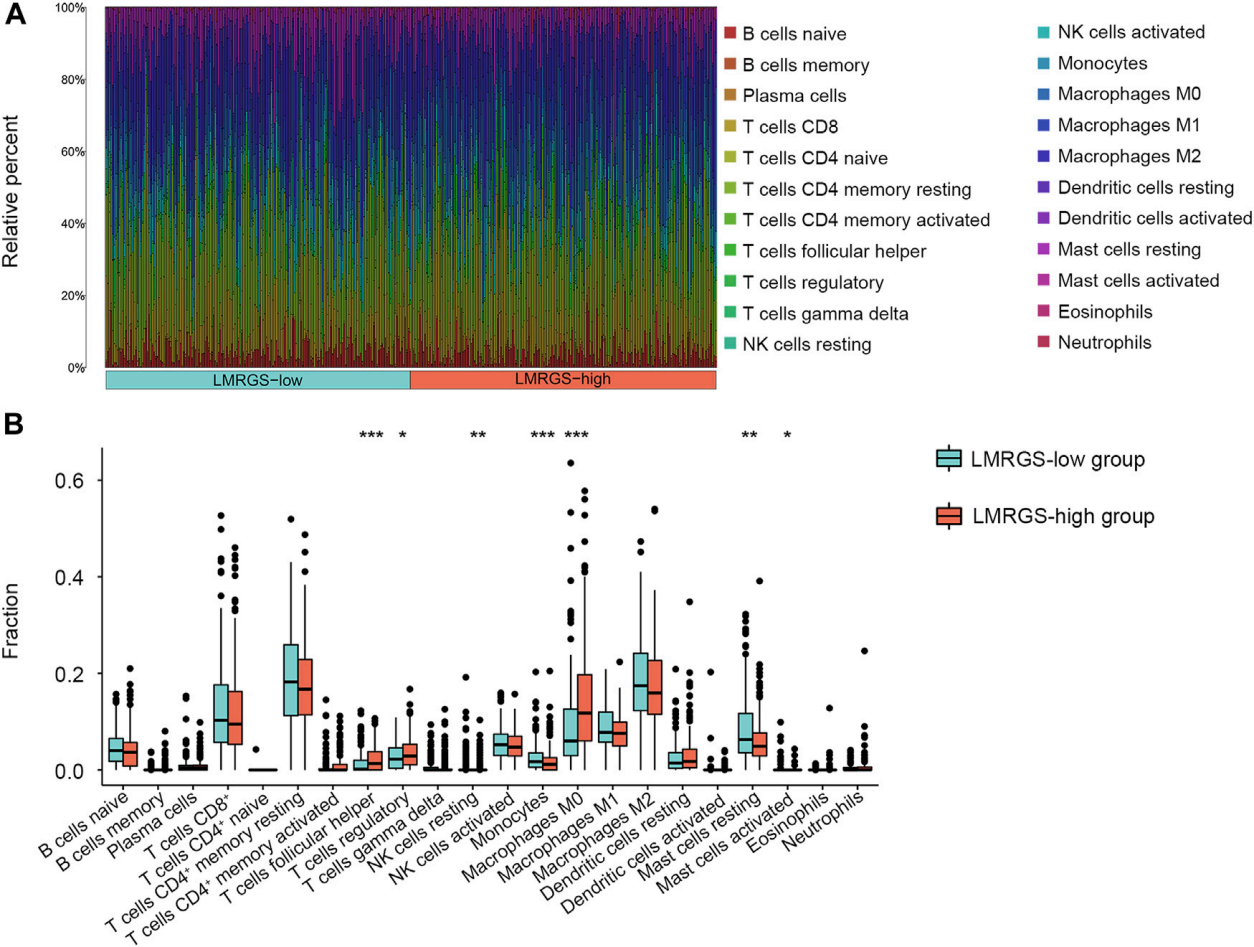
Features of immune cell infiltrate in different LMRGS subgroups. **(A)** The heatmap displayed the proportion of immune cell infiltration in each HCC sample. **(B)** Differences in immune cell infiltration between LMRGS-low and LMRGS-high groups. *p < 0.05, **p < 0.01, ***p < 0.001.

**FIGURE 9:**
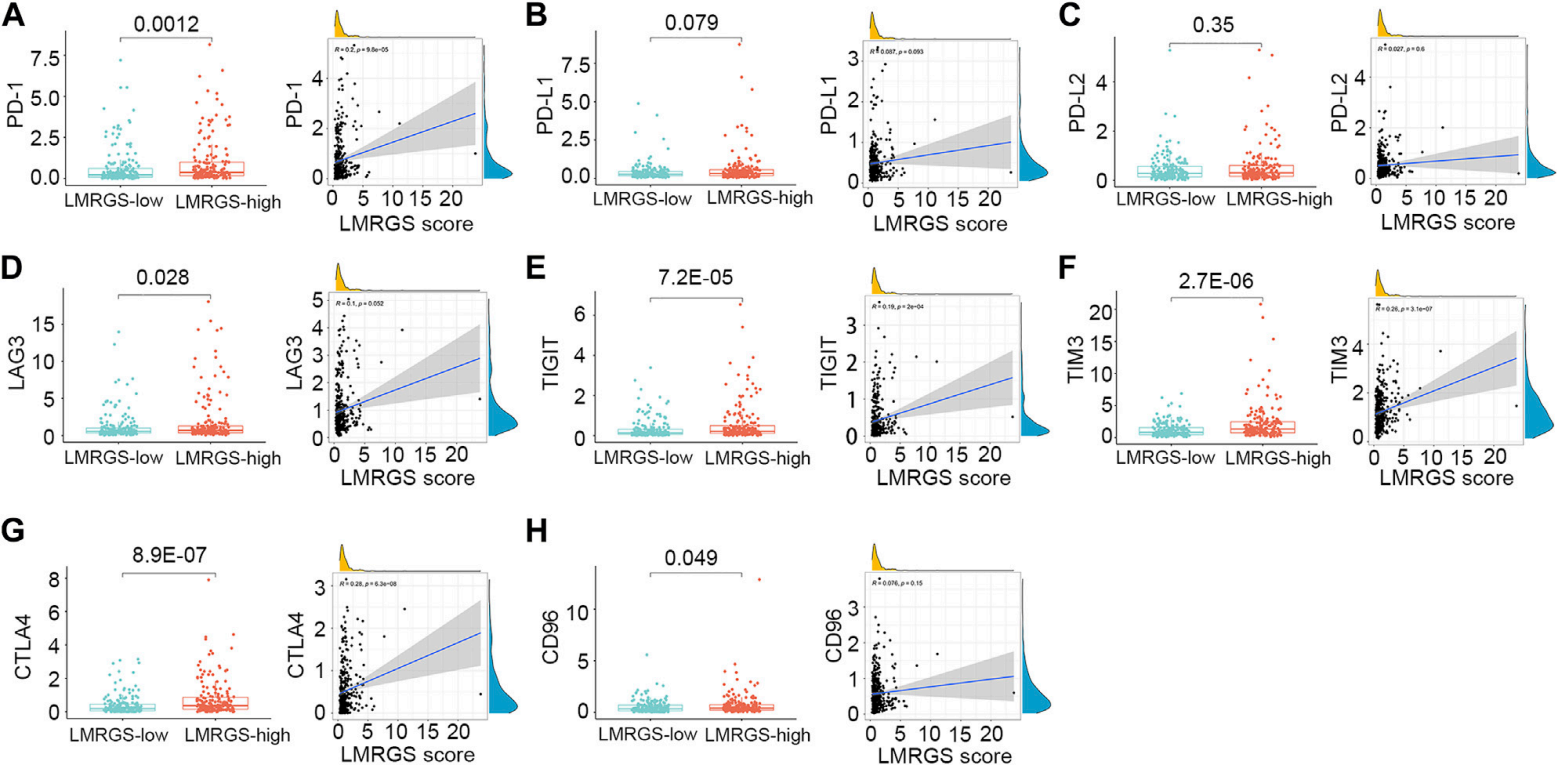
Correlation of LMRGS score with immune checkpoints. **(A)** PD-1. **(B)** PD-L1. **(C)** PD-L2. **(D)** LAG3. **(E)** TIGIT. **(F)** TIM3. **(G)** CTLA4. **(H)** CD96.

To comprehensively analyze the immune microenvironment, we used the CIBERSORTx to calculate the infiltration degree of 22 immune cells. The immune landscape of TCGA-HCC samples was shown in Figure 8A. By comparing the immune cell profiles, we found that follicular helper T (Tfh) cells, regulatory T cells (Tregs), and M0 macrophages were significantly increased in the LMRGS-high group. On the contrary, resting NK cells, monocytes, resting mast cells, and activated mast cells infiltrated more in the LMRGS-low group (Figure 8B). Apart from immune cells, we further explored the correlation of immune molecular and the LMRGS score. In our results, the LMRGS score was positively associated with the expression of immune checkpoints, including PD-1, CTLA4, LAG3, TIM3, and TIGIT (Figure 9).

### GSEA of the LMRGS

**FIGURE 10:**
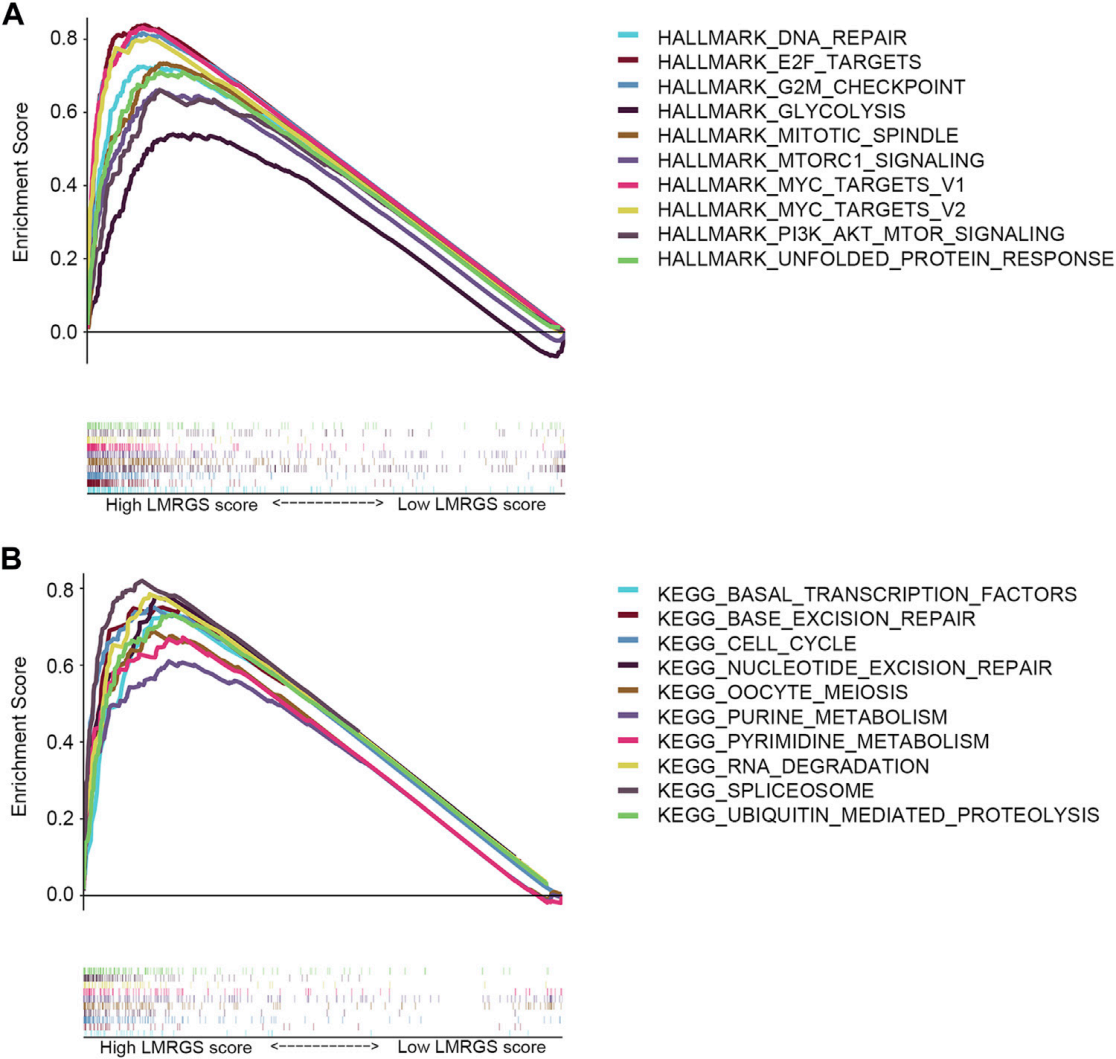
GSEA of LMRGS-low and LMRGS-high groups. **(A)** Enrichment results of hallmark. **(B)** Enrichment results of signaling pathways.

To explore the molecular mechanisms involved in the LMRGS, GSEA was used to analyze the TCGA cohort. Enrichment results of hallmark revealed that DNA repair, E2F targets, G2M checkpoint, glycolysis, mitotic spindle, mTOR signaling, MYC targets, and unfolded protein response were activated by the LMRGS-high group (Figure 10A). Besides, the LMRGS also participated in regulating the transcription factors, DNA repair, cell cycle, and metabolism-related signaling pathways (Figure 10B).

## Discussion

Despite some advances in diagnosis and treatment, HCC is still cancer with high morbidity and mortality (Forner et al., 2018). As inflammation-driven cancer, there is an intricate interplay between the TME and HCC development (Ringelhan et al., 2018). Increasing evidence indicates that metabolic changes of tumors can sculpt their microenvironment, and then the remodeled TME confer a growth advantage to tumor cells (Dimri et al., 2020; Li et al., 2021). Aerobic glycolysis is a vital hallmark of tumor metabolic reprogramming. Glucose is not completely oxidized but metabolized to produce lactate, even in the presence of oxygen (Palsson-McDermott and O'Neill, 2013). Recently, some studies have reported that there is lactate accumulation in tumors (Yu et al., 2021). Lactate is now considered an essential energy substance for tumor metabolism and plays an indispensable role in restructuring the TME (Certo et al., 2021). Hence, we constructed a novel LMRGS based on LMRGs in this study. The results suggested that the LMRGS was an independent prognostic factor for OS. In addition, the LMRGS proved to have substantial value for predicting the TME in HCC.

The LMRGS was composed of six crucial genes, including FKTN, PDSS1, PET117, PUS1, RARS1, and RNASEH1. FKTN participates in protein glycosylation modification (Kanagawa et al., 2016). A study of gastric cancer indicated that higher FKTN expression is associated with tumor progression, which may be due to the protein encoded by FKTN promoting the interaction between tumor cells and the extracellular matrix (Oo et al., 2016). PDSS1 is the critical enzyme in CoQ10 biosynthesis, mediating metabolism and mitochondrial function. The mutation of PDSS1 has an impact on ATP production and oxidative stress (Mollet et al., 2007). As for PET117, mainly distributed in the mitochondrial matrix, it is related to oxidative phosphorylation *via* influencing the biogenesis of cytochrome c oxidase (Vidoni et al., 2017). PUS1 involves in the structural modification of mRNA and is correlated with mitochondrial disorders (Carlile et al., 2019). RARS1 fusion with MAD1L1 has been reported to stimulate the FUBP1/c-Myc signaling pathway, inducing tumorigenesis in nasopharyngeal carcinoma (Zhong et al., 2018). RNASEH1 plays a vital role in maintaining the stability of mitochondrial DNA under oxidative stress (Renaudin et al., 2021). However, the role of six essential genes remains unclear in HCC. To clarify the regulation mechanism of these crucial genes, we performed co-expression analysis between transcription factors and six genes. A total of 52 co-expressed transcription factors were identified, and their functions were mainly reflected in chromatin remodeling and histone modification.

Genomic alterations are the main intrinsic drivers of tumor heterogeneity (Müller et al., 2020). To further understand the molecular features, we compared the gene mutations in different LMRGS groups. As suggested by the results, missense mutation was the most common type of mutations. In the LMRGS-high group, the TP53 gene had the highest mutation rate, while CTNNB1 and TTN were the most frequently mutated genes in the LMRGS-low group. TP53 is not only playing a central role in response to genotoxic stress but also in regulating metabolic homeostasis (Levine, 2020). Increasing evidence reveals the critical functions of TP53 in cellular metabolism (Wang et al., 2018; Kim et al., 2019). The dysfunction of p53 protein encoded by TP53 affects the tumor initiation and progression by mediating the metabolism of tumor cells (Lonetto et al., 2019). The poor prognosis of the LMRGS-high group could be due to TP53 hypermutation. A study reported that increased lactate better meets the metabolic needs of tumor cells and thus favors cell proliferation in p53 mutated tumor cells (Boidot et al., 2012). CTNNB1 and TTN also have links with the malignant transformation of liver cells (Jhunjhunwala et al., 2014). However, patients in the LMRGS-low group had lower probabilities of genetic mutations than those in the LMRGS-high group. Based on the gene mutations of the whole genome, the TMB of every patient was calculated. We found that patients with high TMB and high LMRGS scores had the worst clinical outcomes, which might be because of the genome instability caused by the high TMB (Ferguson et al., 2015).

Complex TME influences tumor progression and response to treatment. There were great differences in the TME between the two LMRGS subgroups, especially in the tumor immune microenvironment. The two groups showed different immune function statuses, including cytolytic activity, MHC class I expression, and IFN response. Cytolytic activity of immune cells reflects the ability to kill tumor cells. Transcriptome hypomethylation of CD8^+^ T cells activates cytolytic activity and effector function, which in turn enhances anti-tumor responses (Loo Yau et al., 2021). In HCC, patients with a high cytolytic activity score have favorable TME and more robust immunogenicity, resulting in better prognoses (Takahashi et al., 2020). Increased expression of MHC class I with high T cell infiltration benefits the prognosis of patients with liver metastases from colon cancer (Turcotte et al., 2014). In our analysis, MHC class I expression was higher in patients with high LMRGS scores. Consequently, the impact of MHC class I expression on the prognosis of HCC patients needs to be further clarified. Besides, the activation of IFN response is an essential link to anti-tumor immunity (Takahashi et al., 2021). As it could be seen, patients in the LMRGS-low group had better anti-tumor immune activity.

Tumor-infiltrating immune cells are one of the most important components in the TME, which can be affected by the lactate level (Certo et al., 2021). Low glucose and high lactate accumulation in the TME have immunosuppressive effects. Under lactate-rich conditions, reducing NAD^+^ to NADH by lactate dehydrogenase (LDH) leads to blocked production of GAPDH and PDGH, which in turn impairs effector T cell proliferation dependent on post-GAPDH glycolytic intermediates (Quinn et al., 2020). Tregs have inhibitory effects on immune response and antigen activation, facilitating cancer progression. Increased aerobic glycolytic activity creates a lactate-enrich microenvironment that favors Tregs survival and contributes immunosuppressive functions (Wang et al., 2017). Moreover, elevated lactate levels in the TME can supply potential nutrition to Tregs, which is due to lactate reversal to pyruvate and NADH in the presence of LDH (Lochner et al., 2015). A study suggested that inhibiting glycolysis and promoting oxidative phosphorylation recover the differentiation of Tfh cells and reduce inflammatory damage (Dong et al., 2019). Another interesting study found that high lactate accumulation decreases the PH of the microenvironment, then promotes NK cell apoptosis and inhibits its natural killer function (Harmon et al., 2019). B cells are of great significance in humoral immune responses through antibody production. Altered intra- and extracellular metabolic signaling can affect the immune regulatory function of B cells (Rosser and Mauri, 2021). Monocytes and mast cells play a vital role in regulating immune responses, and they can alter the TME toward anti-tumor immunity when fully triggered (Guilliams et al., 2018; Dudeck et al., 2019). In addition, macrophages have two central polarization states, including M1 and M2. Different TME leads M0 macrophages polarization to different states, resulting in very opposed effects. M1 macrophages polarization contributes to the immunity against the tumor, while M2 macrophages promote cancer progression and treatment resistance (Chen et al., 2021). Lactate derived from tumors leads to M2 macrophages polarization *via* activating the mTORC2 and ERK signaling pathways (Zhang et al., 2021). The results of our study were consistent with these conclusions. The infiltration levels of B cells, NK cells, monocytes, and mast cells were higher in the LMRGS-low group. Conversely, Tfh cells, Tregs, and M0 macrophages were more abundant in the LMRGS-high group. The results indicated that the immune cells of patients in the LMRGS-high group were affected by lactate metabolism, so the TME was more inclined to an immunosuppressive state.

Apart from the accumulation of immune cells that negatively regulate immune activity, the immunosuppressive TME is also associated with the up-regulated expression of inhibitory immune checkpoints (Sangro et al., 2021). We further explored the differences in the expression of inhibitory molecules between the LMRGS subgroups. In the LMRGS-high group, inhibitory immune checkpoint expressions were significantly higher, including PD-1, CTLA4, LAG3, TIM3, TIGIT, and CD96. In addition, the LMRGS score was positively correlated with PD-1, CTLA4, TIM3, and TIGIT. Recently, immunotherapy targeting inhibitory immune checkpoints has shown promising efficacy in treating advanced HCC (Yau et al., 2020). The expression level of the immune checkpoint is the predictive biomarker of immunotherapy response. From our results, we speculated that patients with high LMRGS scores might gain more benefit from immunotherapy. Besides, TMB associated with neoantigen production is an essential factor in driving anti-tumor immunity. High TMB increases the efficiency of stimulating host immune response (Shum et al., 2021). In our study, HCC patients with high LMRGS scores had high expression of inhibitory immune checkpoints and high TMB. Thus, the LMRGS might have a good value for precisely predicting which patients could respond to immunotherapy.

This study developed a novel LMRGS to predict the prognosis and TME in HCC. Notably, there are certain limitations in the present study. Firstly, the specific molecular functions of six genes involved in the LMRGS remain unclear. There need further experiments to elucidate the role of genes in HCC. Secondly, the LMRGS was constructed and validated using the retrospective data. In the future, we need to carry out multicenter prospective studies to validate the clinical value.

In summary, our study constructed a novel LMRGS with a high value for predicting prognosis and reflecting the TME in HCC. The LMRGS was closely associated with clinical outcomes and was an independent prognostic indicator. In addition, patients with different LMRGS scores had different TME statuses, including infiltration degree of stromal cells and immune cells, immune activity, and expression of immune checkpoints. Thus, the LMRGS was a promising biomarker to speculate molecular and immune features in HCC, which might provide new therapeutic strategies for HCC treatment.

## Data Availability

Publicly available datasets were analyzed in this study. This data can be found here: https://portal.gdc.cancer.gov/, https://dcc.icgc.org/releases/current/Projects/LIRI-JP.

## Abbreviations

HCC, Hepatocellular carcinoma; LMRGs, Lactate metabolism-related genes; LMRGS, Lactate metabolism-related gene signature; TMB, Tumor mutation burden; TME, Tumor microenvironment.
