# Supplementary material for: A Novel Lactate Metabolism-Related Gene Signature for Predicting Clinical Outcome and Tumor Microenvironment in Hepatocellular Carcinoma
Source: Front Cell Dev Biol. 2022 Jan 3;9:801959. doi: 10.3389/fcell.2021.801959 (PMC8762248; doi:10.3389/fcell.2021.801959)
Supplement: Supplementary file 1 [file DataSheet1.DOCX]

**SUPPLEMENTARY MATERIALS**

**
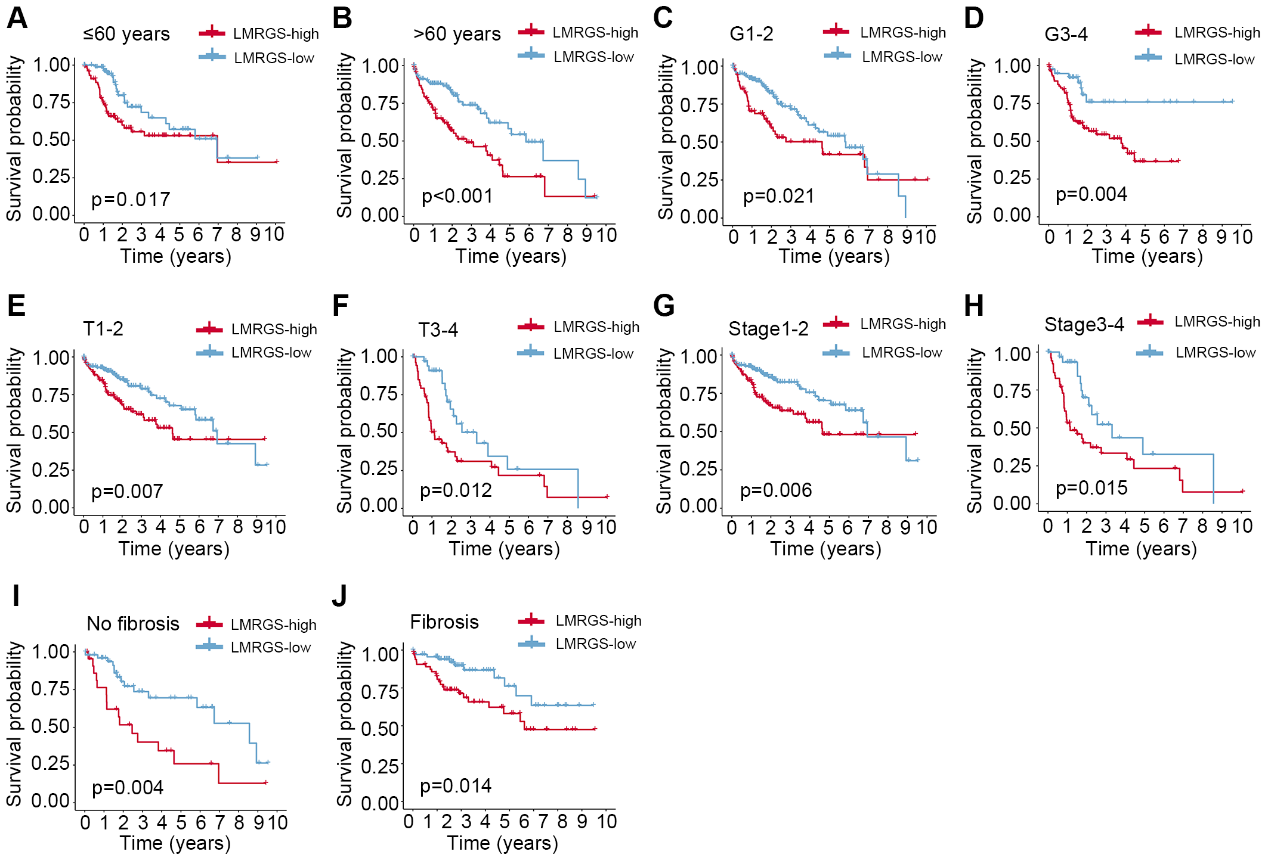
**

**SUPPLEMENTARY FIGURE 1** Survival analyses in HCC patients classified according to age, grade, tumor staging, clinical staging, and fibrosis. (A) ≤ 60 years. (B) > 60 years. (C) Grade 1–2. (D) Grade 3–4. (E) Tumor staging 1–2. (F) Tumor staging 3–4. (G) Clinical staging 1-2. (H) Clinical staging 3-4. (I) No fibrosis. (J) Fibrosis.


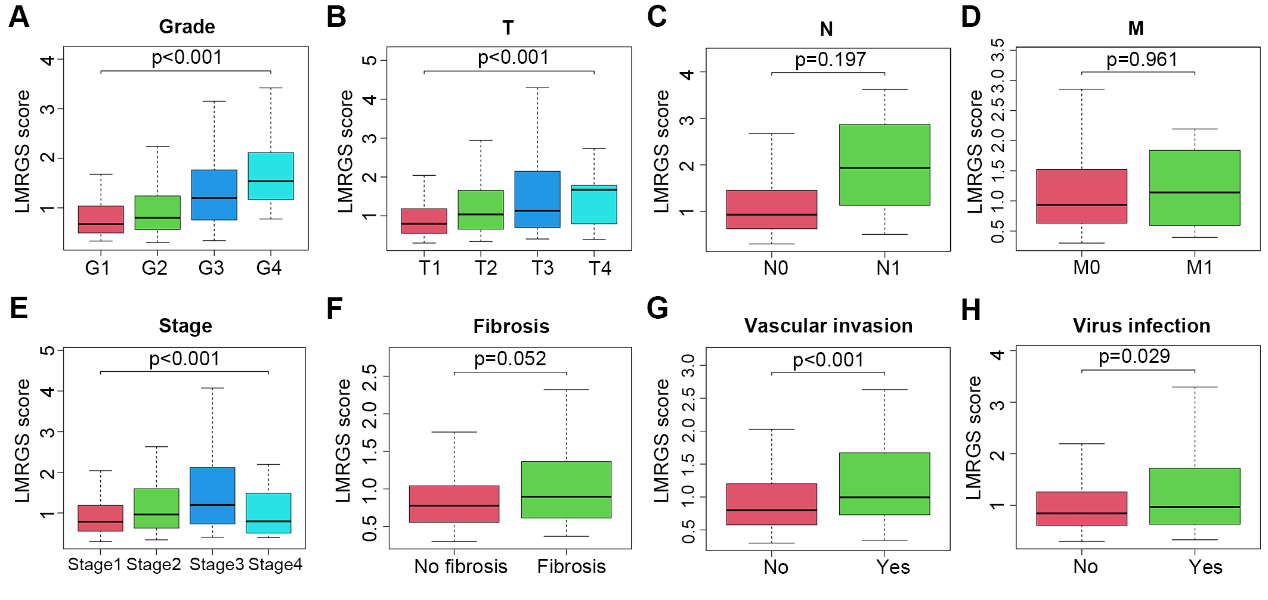


**SUPPLEMENTARY FIGURE 2** The association of LMRGS score with clinicopathological factors. (A) Grade. (B) Tumor staging. (C) Node staging. (D) Metastatic staging. (E) Clinical staging. (F) Fibrosis. (G) Vascular invasion. (H) Virus infection.

**
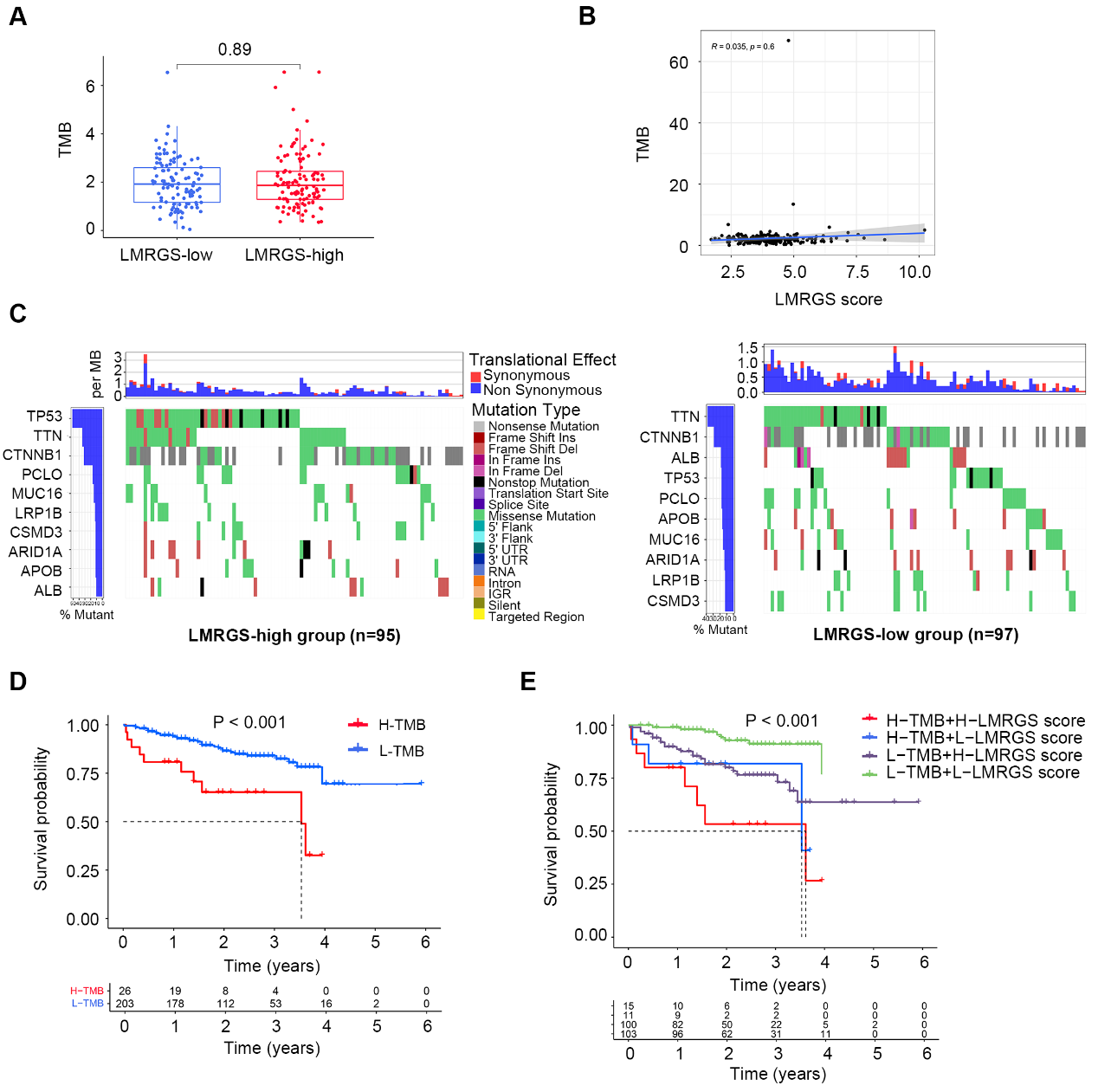
**

**SUPPLEMENTARY FIGURE 3** Tumor mutation characteristics in ICGC cohort. (A) The differences of TMB in LMRGS-low and LMRGS-high groups. (B) The association of TMB with LMRGS score. (C) Top 10 mutated genes in different LMRGS subgroups. (D) KM survival analysis of TMB. (E) Effects of the LMRGS score combined with TMB on the overall survival.

**
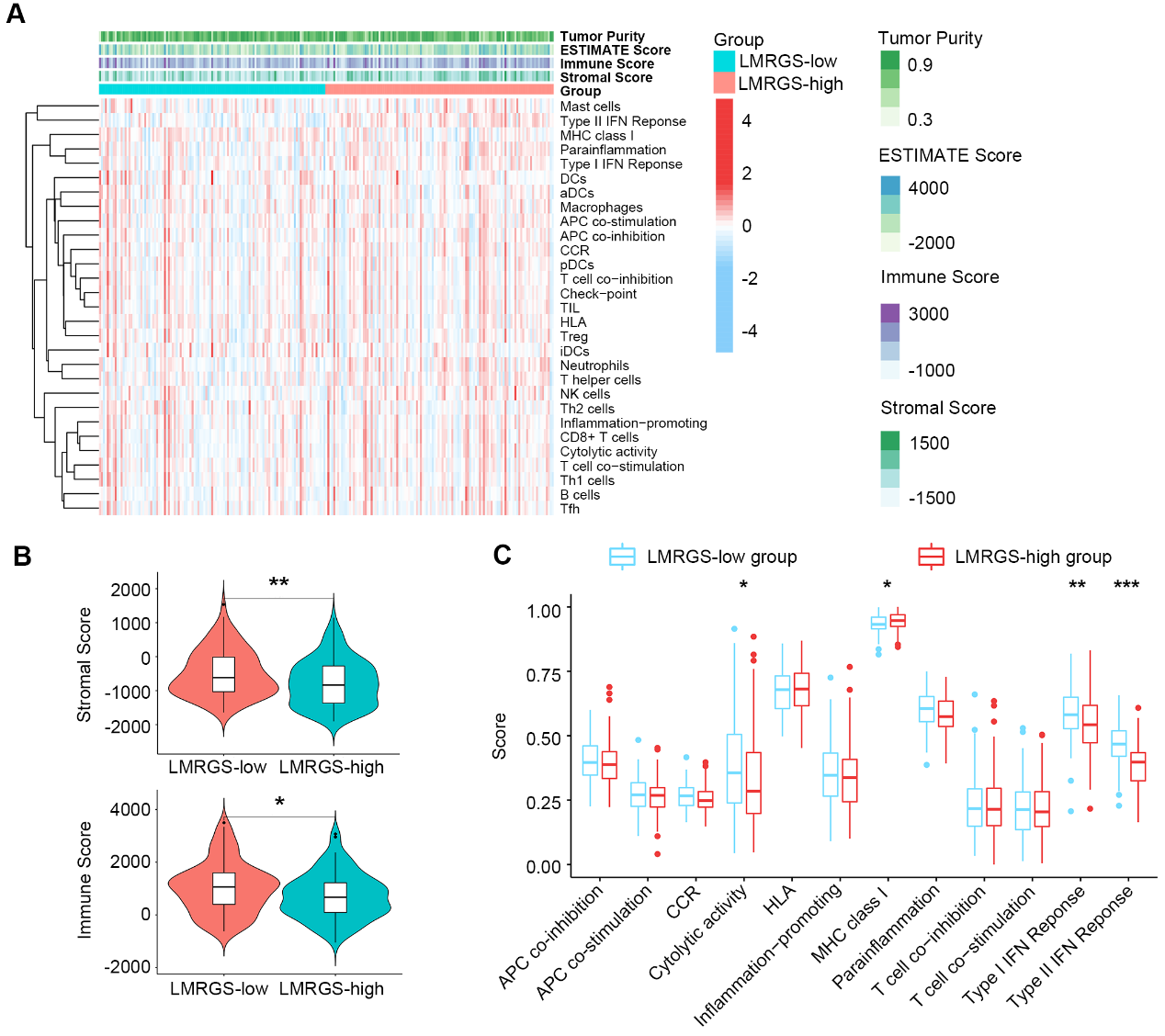
**

**SUPPLEMENTARY FIGURE 4** The landscape of TME in ICGC cohort. (A) Stromal score and immune activity of all HCC samples. (B) The violin plot showed the difference in stromal scores and immune scores between LMRGS-low and LMRGS-high groups. (C) Differences in immune function between the two subgroups. * P < 0.05, ** P < 0.01, *** P < 0.001.
